# Glucose Fluctuation Inhibits Nrf2 Signaling Pathway in Hippocampal Tissues and Exacerbates Cognitive Impairment in Streptozotocin-Induced Diabetic Rats

**DOI:** 10.1155/2024/5584761

**Published:** 2024-01-19

**Authors:** Haiyan Chi, Yujing Sun, Peng Lin, Junyu Zhou, Jinbiao Zhang, Yachao Yang, Yun Qiao, Deshan Liu

**Affiliations:** ^1^Shandong University of Traditional Chinese Medicine, Jinan, Shandong, China; ^2^Department of Endocrinology, Weihai Municipal Hospital, Cheeloo College of Medicine, Shandong University, Weihai, Shandong, China; ^3^Department of Traditional Chinese Medicine, Qilu Hospital of Shandong University, Jinan, Shandong, China; ^4^Department of Neurology, Weihai Municipal Hospital, Cheeloo College of Medicine, Shandong University, Weihai, Shandong, China

## Abstract

**Background:**

This research investigated whether glucose fluctuation (GF) can exacerbate cognitive impairment in streptozotocin-induced diabetic rats and explored the related mechanism.

**Methods:**

After 4 weeks of feeding with diets containing high fats plus sugar, the rat model of diabetes mellitus (DM) was established by intraperitoneal injection of streptozotocin (STZ). Then, GF was triggered by means of alternating satiety and starvation for 24 h. The weight, blood glucose level, and water intake of the rats were recorded. The Morris water maze (MWM) test was carried out to appraise the cognitive function at the end of week 12. Moreover, the morphological structure of hippocampal neurons was viewed through HE and Nissl staining, and transmission electron microscopy (TEM) was performed for ultrastructure observation. The protein expression levels of Nrf2, HO-1, NQO-1, Bax, Bcl-2, and Caspase-3 in the hippocampal tissues of rats were measured *via* Western blotting, and the mRNA expressions of Nrf2, HO-1, and NQO-1 were examined using qRT-PCR. Finally, Western blotting and immunohistochemistry were conducted to detect BDNF levels.

**Results:**

It was manifested that GF not only aggravated the impairment of spatial memory in rats with STZ-induced type 2 DM but also stimulated the loss, shrinkage, and apoptosis of hippocampal neurons. Regarding the expressions in murine hippocampal tissues, GF depressed Nrf2, HO-1, NQO-1, Bcl-2, and BDNF but boosted Caspase-3 and Bax.

**Conclusions:**

GF aggravates cognitive impairment by inhibiting the Nrf2 signaling pathway and inducing oxidative stress and apoptosis in the hippocampal tissues.

## 1. Introduction

As a severe progressive chronic metabolic disorder, diabetes mellitus (DM) has become a global epidemic and public health problem causing significant medical and social burdens. The number of DM patients has been increased significantly over the past few years mainly because the incidence of type 2 DM (T2DM) is rising, impacting up to 14% of people aged above 60 years old [1]. As estimated by the International Diabetes Federation (IDF), there were about 41,600 cases of newly diagnosed T2DM in 2021 worldwide, and 7,079 out of 100,000 people will suffer from the disease as of 2030 [2] Moreover, 239.7 million (44.7%) adults (20-79 years old) had undiagnosed DM, thus missing the timely treatment [3]. Globally, the DM-related health expenditures were estimated at 966 billion USD in 2021, which are projected to reach 1,054 billion USD by 2045. DM can result in over 1 million deaths every year, becoming the ninth leading cause of death [4, 5].

T2DM is strongly associated with many acute and chronic complications, especially those of the brain and nervous system, which not only induce a marked decline in the quality of life but also cause death and disability in severe cases. Due to the constant complaint of memory loss and inattentiveness by patients, DM complications related to the central nervous system have been clarified by clinicians and researchers for more than 100 years. According to clinical and epidemiological studies, T2DM increases the risk of impairing cognitive abilities such as attention, memory, concentration, executive function, information processing, visual-spatial ability, and psychomotor speed, thereby prominently reducing the quality of survival. Despite normal cognition in daily life, DM patients in all age groups have more subclinical cognitive deficits than people without DM, as revealed by psychological tests. Moreover, the risk of developing dementia is raised under the prediabetic metabolic condition; that is, the risk is elevated in a “dose-dependent” manner [6]. Meta-analysis results have uncovered that the incidence of dementia rises distinctly by 30-100 percent in DM patients [7], and such patients are also susceptible to severer dementia [8]. Therefore, it is crucial to explore the possible risk factors and underlying mechanisms of cognitive impairment in the case of DM.

The brain is the most vulnerable to glucose metabolism disorder because its energy is almost entirely derived from glucose, and it is primarily driven by neuronal signaling, biosynthesis, and neuroprotection. As the brain develops into the adult level after the age of 20 years old, it consumes approximately 20% of the body's energy although it merely accounts for 2% of the body weight [9]. The potential damage to the brain that is in the most active state during cognitive development may cause irreparable injuries to cells and structure [10, 11], which may persist into adulthood. Glucose metabolism disorder in diabetic patients can be characterized by constant hyperglycemia, fluctuating hyperglycemia and hypoglycemia. Glucose fluctuation (GF) refers to a volatile condition where the glucose level varies between the peak and valley. Extreme glucose differences may alter the expressions of NRG1, ErbB receptor subtypes, Syntaxin 1, and OLIG1, which are associated with regeneration damage, synaptic dysfunction, demyelination, cognitive impairment, and anxiety [12].

It has been evidenced by brain imaging techniques that glucose metabolism disorder is a critical player in brain changes [13–15], indicating the significance of optimal control. However, the relation of GF to cognitive impairment in the case of DM is rarely researched, and the specific mechanism remains to be elaborated. The present study intends to elucidate the effects of GF on the cognitive function of streptozotocin- (STZ-) induced diabetic rats and investigate the pathophysiological changes besides the potential mechanisms.

## 2. Materials and Methods

### 2.1. Animals and Ethical Statement

Male Sprague-Dawley rats (6 weeks old, weighing 160-200 g) were purchased from the Experimental Animal Center of Shandong University of Traditional Chinese Medicine. The rats underwent 1-week adaptive breeding in a new environment prior to the experiments, and they stayed under specific pathogen-free conditions (temperature: 20-23°C, humidity: 50 ± 5%, and a 12 h/12 h light/dark cycle) with free access to water during the experiments. All experiments and protocols were in conformance with the regulations of the Institutional Animal Care and Use Committee, Shandong University of Traditional Chinese Medicine (SDUTCM20221027001).

### 2.2. Materials

Rat models of T2DM were established using the methods in previous literature [16, 17]. After 4 weeks of raising with high-sugar and high-fat diets (66.5% general fodder + 20%sugar + 10%fat + 2.5%cholesterol + 1% sodium cholate), the rats were administered with STZ (35 mg/kg, Sigma Chemical, St. Louis, MO, USA) dissolved in citrate buffer (0.09 mol/L, pH 4.5) through intraperitoneal injection. The rats in the control (Con) group were injected with vehicle (sodium citrate buffer, pH 4.5) in an equal volume. Three days later, the DM model was successfully constructed in rats with blood glucose levels exceeding 300 mg/dL.

### 2.3. Experimental Design

As per the completely random double-blind method, the rats were assigned into two groups, namely, the Con group (*n* = 10) and the diabetic group (*n* = 20). Rats in the Con group were fed with a common diet, whereas those in the diabetic group ate high-sugar and high-fat diets throughout the experiment. Next, the diabetic rats were randomly allocated into DM and GF groups, with 10 rats in each group. Rats in the Con and DM groups were fed with adequate food, while those in the GF group alternatingly suffered 24 h of satiety and 24 h of starvation to induce GF, as described in previous studies [18–20].

**Figure 1:**
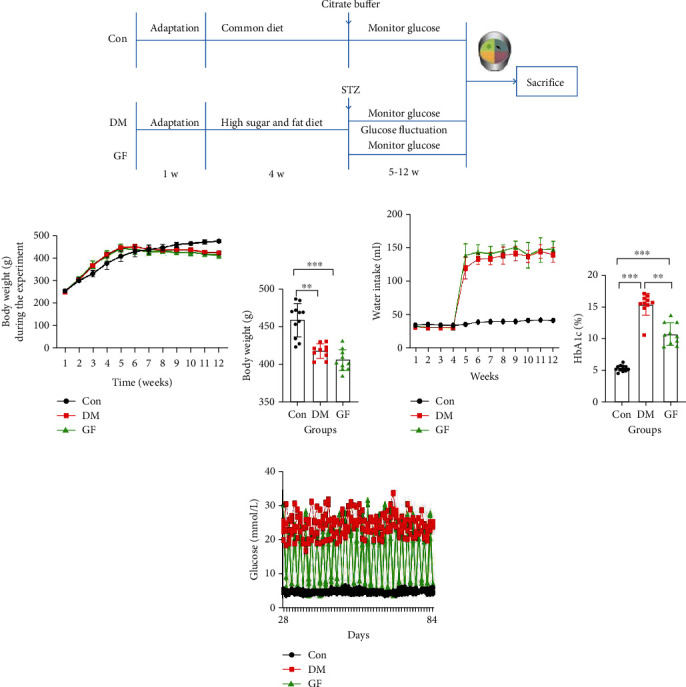
Schematic overview of the experimental procedure, body weight, water intake, HbA1c, and glucose levels. (a) Schematic overview of the experimental procedure. (b) The trend chart of body weight during the experiment. (c) At the end of the experiment, the body weight of the Con group was higher than that of the DM and GF groups. While the body weight of the GF group was slightly lower than that of the DM group, the difference was not statistically significant (*p* = 0.063). (d) Water intake during the experiment. (e) HbA1c in the three groups. (f) Levels of glucose. *p* values were calculated with ANOVA and Tukey's *post hoc* test (^∗∗^*p* < 0.01, ^∗∗∗^*p* < 0.001).

The cognitive function was evaluated using the Morris water maze (MWM) test at the end of week 12. Afterward, the rats were sacrificed, and their brain samples were snap-frozen in liquid nitrogen and preserved at -80°C, or they were directly preserved in 4% paraformaldehyde for histological analysis. The experimental process in this study is displayed in Figure 1(a).

### 2.4. Body Weight, Water Intake, and Glucose Monitoring

Water intake was weekly measured together with body weight. An ACCU-Chek glucose meter (Roche) was employed to acquire the glucose level at 8 : 00 am every day. Glycosylated hemoglobin (HbA1c) content was determined through a commercially available kit (NycoCard, Oslo, Norway) at the end of the experiment.

### 2.5. MWM Test

The spatial learning and memory capacities of rats were assessed *via* the MWM test described in prior literature [21, 22]. MWM was a round stainless steel pool (diameter: 180 cm, height: 60 cm), which was divided into four equal quadrants and surrounded by a dark black curtain to prevent confounding visual cues. A platform (4.5 cm in diameter) was placed at a selected location and submerged to 2 cm below the water surface. In each trial, the duration from a rat being placed into the water to finding out and climbing onto the platform was recorded as the escape latency (EL). The rat was allowed to stay on the platform for 10 s after reaching the hidden platform. If the rat failed to find out the platform within 120 s, it would be directed onto the platform for 10 s and then put back into the home cage. The hidden platform in the target quadrant was removed after 5 days. A probe trial was performed on day 6, during which the rat was allowed to swim freely for 90 s, and its performance was recorded by video tracking software (SMART V2.5, Spain).

### 2.6. Hematoxylin-Eosin (HE) Staining

All rats were anesthetized with isoflurane and decapitated subsequent to behavioral tests. The brain tissues of the rats were collected, immediately fixed in 4% paraformaldehyde (Biosharp Life Sciences, BL539A), dehydrated with gradient ethanol, and embedded in paraffin. Then, the tissues were prepared into slides with a thickness of 5 *μ*m and subjected to standard HE staining. Finally, the morphology of hippocampal neurons was observed under Pannoramic DESK NanoZoomer S60 (C13210-01, HAMAMATSU, Co., Ltd.), followed by analysis.

### 2.7. Nissl Staining

Nissl staining was performed to observe Nissl bodies and neuronal damage in the hippocampal CA1, CA3, and DG regions of rats. Specifically, hippocampal sections were prepared from paraffin-embedded rat brain tissues using a slicer (RM2235, Leica, Germany). Next, the sections were routinely dewaxed in water, and Nissl staining was conducted in accordance with the standard protocol [23, 24]. Finally, the sections were sealed with neutral balsam, imaged with Pannoramic DESK NanoZoomer S60 (C13210-01, HAMAMATSU, Co., Ltd.), and analyzed.

### 2.8. Transmission Electron Microscopy (TEM)

For TEM analysis, the tissue blocks from the hippocampal CA1 region (1 mm × 1 mm × 1 mm) were fixed with 2.5% glutaraldehyde for 2-4 h at 4°C and then posttreated in 1% osmium tetroxide for 1.5 h in the dark. Subsequently, the samples were dehydrated in gradient ethanol and infiltrated by acetone and embedding medium Epon 812 (SPI). After complete polymerization, the samples were observed under a transmission electron microscope (Hitachi, HT-7800, Japan).

### 2.9. Western Blotting Assay

Fresh hippocampal tissues were acquired from rats and made into homogenate, followed by lysis on ice with RIPA lysate as well as phosphatase and protease inhibitors at 100 : 1. Western blotting was performed as described in previous literature [25, 26]. Rabbit anti-Nrf2 (1 : 1000, TA0639S, Abmart), anti-HO-1 (1 : 1000, ab68477, Abcam), anti-NQO-1 (1 : 10000, ab80588, Abcam), anti-Bax (1 : 1000, #14796S, CST), anti-Bcl-2 (1 : 1000, ab196495, Abcam), anti-Caspase-3 (1 : 1000, #9662, CST), anti-Cleaved Caspase-3 (Asp175) (1 : 1000, #9661, CST), and anti-BDNF (1 : 1000, ab226843, Abcam) primary antibodies, as well as horseradish peroxidase- (HRP-) conjugated goat anti-rabbit IgG (1 : 20000) secondary antibody, were applied. The blots were visualized using ECL reagent kits, and ImageJ software was utilized to quantify the optical density of the lanes.

### 2.10. Quantitative Real-Time Polymerase Chain Reaction (qRT-PCR) Assay

The qRT-PCR assay was implemented according to prior literature [27]. To be specific, RNAprep Pure Micro Kit (Aidlab, Beijing, China) was adopted to extract total RNAs from the hippocampus, which were reversely transcribed into cDNA as per the standard protocol. Then, amplification was executed with the following parameters: 95°C for 120 s plus 40 cycles of 95°C for 10 s and 60°C for 30 s. The gene expression levels were calculated based on the 2^−ΔΔCt^ method. Each sample was analyzed in triplicate, and the relative mRNA expression was determined after normalization to *β*-actin. The relative mRNA expression in the Con group (target mRNA/*β*-actin ratio) was set to 100%, and the mRNA expressions in other groups were converted to fold changes through comparing with that in the Con group. The utilized primer sequences are listed in Table 1.

### 2.11. Measurement of Oxidative Stress Parameters

The malondialdehyde (MDA) level and superoxide dismutase (SOD) activity in the hippocampal tissues were evaluated using commercially available kits (A003-1 for MDA, A001-3 for SOD, Nanjing Jiancheng Bioengineering Institute, Nanjing, China). The experiment was performed in accordance with the manufacturer's instructions. A microplate reader (Tecan Infinite M200, Shandong, China) was employed to detect the absorbance at 532 nm and 450 nm. All samples (*n* = 5 for each group) were examined in triplicate to obtain the mean value.

### 2.12. Immunohistochemistry (IHC)

IHC was performed as described in previous research [28]. Briefly, paraffin-embedded rat brain tissues were incubated at 1/500 dilution ratio and 4°C overnight. Then, the tissues were subjected to antigen retrieval in citrate buffer (pH 6.0) for 15 min (Cuisinart Electric Pressure Cooker #EPC-1200, with “high pressure” selected), blocking with endogenous peroxidase containing 3% H_2_O_2_, RT for 30 min, blocking in 1.5% goat serum (diluted by 1× PBS), RT for 30 min, and incubation with the secondary antibody using ABC-HRP Kit (Rabbit IgG at 1 : 200 dilution), followed by 30 min of RT, washing in PBS for 2 × 5 min, and color development with DAB through the chromogen system.

### 2.13. Statistical Analysis

Data were presented as mean ± standard deviation and analyzed using GraphPad Prism software (GraphPad Software Inc., La Jolla, CA, USA). Unpaired Student's *t*-test was carried out to compare data between groups, and one-way ANOVA with Tukey's *post hoc* LSD analysis was implemented for data comparison among multiple groups. *P* < 0.05 implied a difference of statistical significance.

## 3. Results

### 3.1. With the Glucose Level Elevated, the Body Weight of Rats Exhibited a Downward Trend, While the Water Intake Was Increased Significantly in the DM and GF Groups

The glucose in the DM group was maintained at a high level, while that in the GF group fluctuated greatly. During the experiments, one rat in the DM group died of pulmonary infection at week 10, and one in the GF group died of tail infection at week 9. The body weight of rats was increased rapidly in the DM and GF groups, but it presented a decreasing trend from week 6. The body weight in the Con group maintained an upward trend during the experiments (Figures 1(b) and 1(c)). The water intake of rats was remarkably raised in the DM and GF groups from week 4, while it was changed little in the Con group (Figure 1(d)). The Con group had the lowest HbA1c level, and the DM group manifested a higher HbA1c level than the GF group (Figure 1(e)). During the experiments, the blood glucose from the caudal vein fluctuated notably in the GF group, whereas it remained stable in the Con and DM groups (Figure 1(f)).

### 3.2. GF Aggravated Cognitive Dysfunction in Diabetic Rats

**Figure 2:**
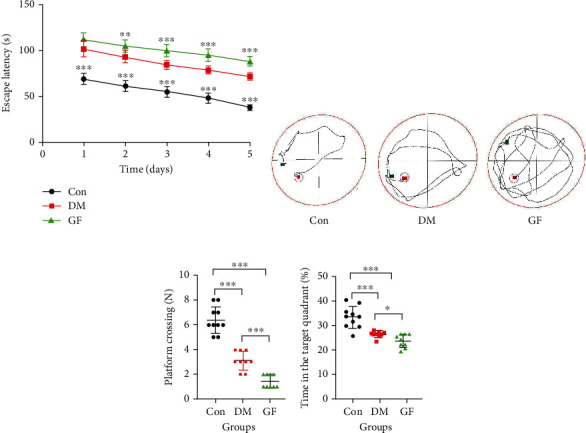
Effect of GF on learning and memory in diabetic rats. (a) Changes of EL during the experiment (*n* = 9 − 10 each group). (b) Representative swimming trajectories of the three groups of rats. (c) Numbers of platform crossing. (d) Time in the target quadrant (%). *p* values were calculated with ANOVA and Tukey's *post hoc* test (^∗^*p* < 0.05, ^∗∗^*p* < 0.01, ^∗∗∗^*p* < 0.001).

The MWM test was executed to assess the cognitive function of rats in each group. On days 1-5, the EL was gradually shortened in all groups. By contrast to the Con group, the DM and GF groups possessed longer EL during days 1-5. However, there was no significant difference in EL between the DM group and the GF group on day 1 (*P* = 0.069), but EL was extended on days 2-5 in the GF group in comparison with that in the DM group (Figures 2(a) and 2(b)). On day 6 of the probe trial, the frequency of platform crossing was decreased in the DM and GF groups, and it was the lowest in the GF group (Figure 2(c)). Besides, the Con group presented a longer time of stay in the target quadrant (%) than the DM and GF groups, and the rats in the GF group stayed in the target quadrant for the shortest time (Figure 2(d)). Behavioral test results indicated that GF could aggravate the impairment of spatial learning and memory capacities in diabetic rats.

### 3.3. Damage and Loss of Hippocampal Neurons in Rats Were Induced by GF

The cognitive function is closely related to the morphological structure of the hippocampus. Therefore, hippocampal neurons were observed by means of HE staining and Nissl staining.

**Figure 3:**
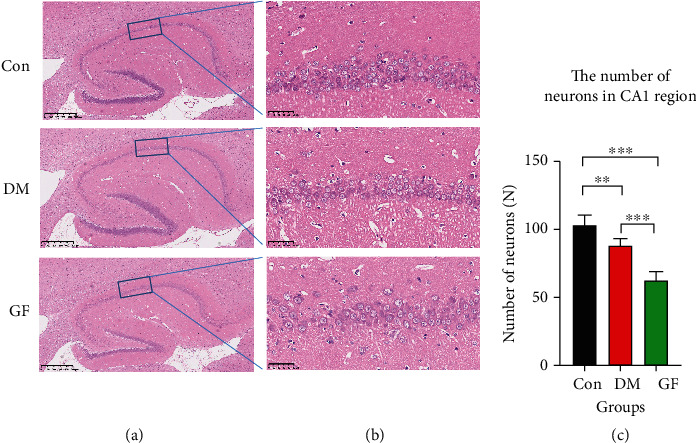
The representative images of HE staining for hippocampal tissue sections in each group of rats. (a) Overall (50x, 500 *μ*m), (b) CA1 region (400x, 50 *μ*m), and (c) the number of neurons in the CA1 region in each group (*n* = 5 each group). *p* values were calculated with ANOVA and Tukey's *post hoc* test (^∗∗^*p* < 0.01, ^∗∗∗^*p* < 0.001).

According to the results of HE staining, the Con group possessed complete tissue structure in the hippocampal CA1 region of rats, with an orderly arrangement of nerve cells, regular appearance, clear envelope, and obvious nuclei. In the DM group, the nerve cells in the hippocampal CA1 region were reduced in number and disorderly arranged, the tissue structure was obviously loosened with irregular appearance, and the envelope was unclear. In the GF group, the above changes in the hippocampal CA1 region were aggravated, a large number of nerve cells were vacuolated, some cells shrank, and the nuclei disappeared. The number of neurons in the DM and GF groups was decreased by contrast to that in the Con group, and it was further lowered in the GF group (Figure 3).

**Figure 4:**
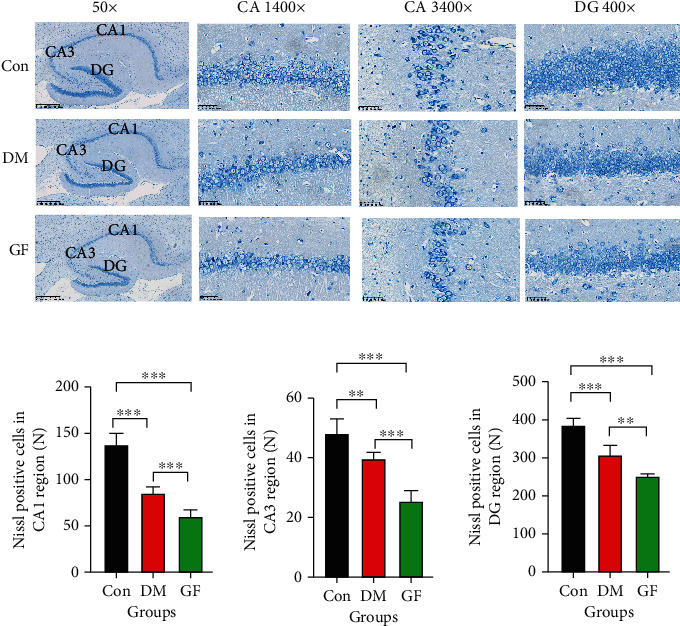
The representative images of Nissl staining for hippocampal tissue sections in each group of rats. (a) Overall (50x, 500 *μ*m), CA1 region (400x, 50 *μ*m), CA3 region (400x, 50 *μ*m), and DG region (400x, 50 *μ*m) of the hippocampus. (b) Comparison of Nissl positive cells between groups (*n* = 5 each group). *p* values were calculated with ANOVA and Tukey's *post hoc* test (^∗∗^*p* < 0.01, ^∗∗∗^*p* < 0.001).

It was discovered through Nissl staining that the Con group manifested abundant neurons and Nissl bodies in the rat hippocampus, with orderly cell arrangement, regular cell morphology, clear outline, and massive nerve cells. The number of nerve cells in hippocampal CA1, CA3, and DG regions declined overtly in the DM and GF groups, with shrunk cells, blurred outline, and evidently decreased Nissl bodies. In comparison to the DM group, the GF group presented a further reduced number of nerve cells (Figure 4).

### 3.4. GF Caused Neuronal Contraction, Lipofuscin Deposition, and Cell Gap Enlargement in the Hippocampal CA1 Region

**Figure 5:**
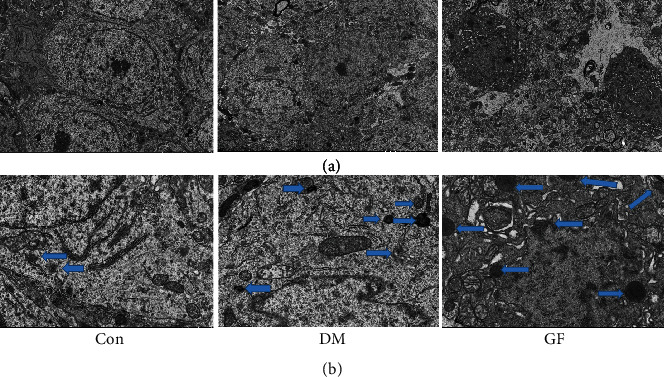
Ultrastructure of hippocampal CA1 region in each group. (a) 3000x (5 *μ*m) and (b) 10000x (1 *μ*m) (*n* = 3 each group). Blue arrows indicated lipofuscin deposited inside the cells.

TEM was carried out to further probe into the neuronal ultrastructure in the hippocampal CA1 region. The Con group had oval hippocampal neurons in an intact and distinct shape, large and round nuclei, and uniform cytoplasm and chromatin. The DM group exhibited serrated edges of cytoplasm and nucleus in a dark color, enlarged intercellular space, and increased lipofuscin. The GF group had hyperpyretic cells which were dark-colored and significantly wrinkled, with irregular nuclei, expanded intercellular space, reduced mitochondrial cristae, and early apoptotic changes (Figure 5).

### 3.5. GF Resulted in Further Inhibition of the Nrf2-HO-1/NQO-1 Signaling Pathway in the Hippocampus of Diabetic Rats

**Figure 6:**
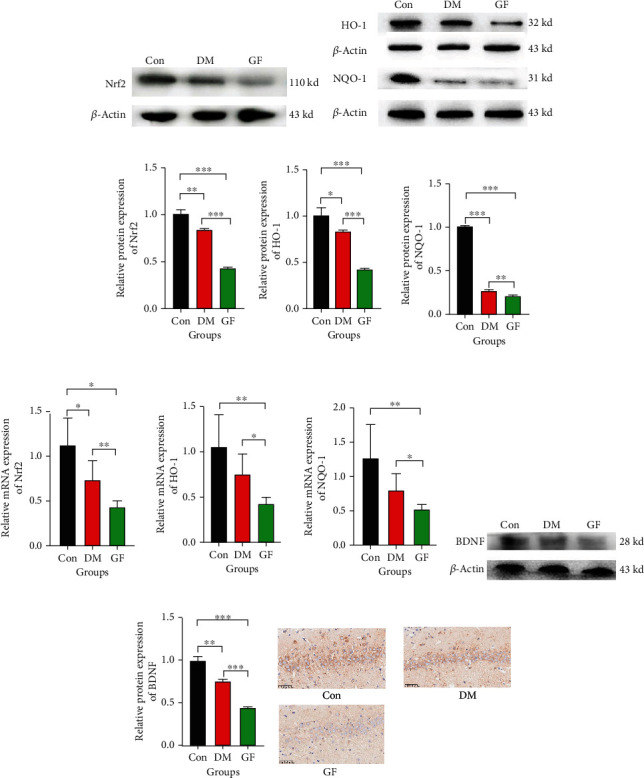
Levels of Nrf2, HO-1, NQO-1, and BDNF in the hippocampus of rats. (a) Western blotting for detecting Nrf2 level in each group. (b) Western blotting for detecting HO-1 and NQO-1 levels in each group. (c) Quantitative analysis of Nrf2, HO-1, and NQO-1 expressions according to the Western blotting results. (d) Comparison of mRNA levels of Nrf2, HO-1, and NQO-1 in hippocampal tissues of three groups. (e) Western blotting for BDNF level in each group. (f) Quantitative analysis of BDNF expression according to the Western blotting results. (g) IHC for the expression of BDNF in the CA1 region (*n* = 5 each group). *p* values were calculated with ANOVA and Tukey's *post hoc* test (^∗^*p* < 0.05, ^∗∗^*p* < 0.01, ^∗∗∗^*p* < 0.001).

For the purpose of validating the signaling pathway through which GF destroys hippocampal neurons, Western blotting and qRT-PCR assays were adopted to examine the expression levels of Nrf2, HO-1, and NQO-1 in rat hippocampal neurons. The results indicated that Nrf2 expression in the hippocampus was inhibited in the DM group and further decreased in the GF group compared with that in the DM group (Figures 6(a) and 6(c)), suggesting that GF can further repress Nrf2 protein. The protein content of HO-1 and NQO-1 showed the same trends (Figures 6(b) and 6(c)). In addition, Nrf2, HO-1, and NQO-1 displayed similar tendencies of mRNA expression levels (Figure 6(d)), hinting that GF is able to suppress the Nrf2-HO-1/NQO-1 signaling pathway.

### 3.6. GF Suppressed BDNF Expression

As a major neurotrophic factor in the brain, BDNF not only regulates synaptic plasticity and important cellular events but also underlies neuronal activity, learning, and memory formation. Further observation of the expression of BDNF protein in the hippocampus demonstrated that DM could inhibit BDNF expression, which was further suppressed by GF (Figures 6(e) and 6(f)). These findings coincided with the GF-triggered decline in learning and memory ability in the MWM test. The IHC results were in line with the Western blotting results (Figure 6(g)).

### 3.7. GF Increased Expressions of Apoptosis-Related Proteins in the Hippocampus of Diabetic Rats

**Figure 7:**
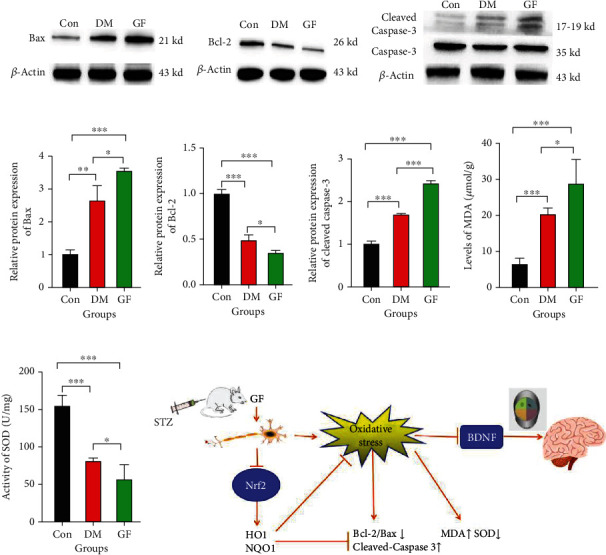
Apoptosis-related proteins in the hippocampus and potential mechanism of cognitive decline induced by GF. (a) Western blotting for Bax in each group. (b) Western blotting for Bcl-2 in each group. (c) Western blotting for Cleaved Caspase-3 in each group. (d) Quantitative analysis of Bax, Bcl-2, and Cleaved Caspase-3 expressions according to the Western blotting results. (e) MDA level in each group. (f) SOD activity in each group. *p* values were calculated with ANOVA and Tukey's *post hoc* test. (g) Potential mechanism of cognitive decline in diabetic rats induced by GF (^∗^*p* < 0.05, ^∗∗^*p* < 0.01, ^∗∗∗^*p* < 0.001).

The TEM results indicated early apoptotic changes of hippocampal neurons in the GF group. Therefore, the expressions of apoptosis-related proteins Bax, Bcl-2, and Caspase-3 were measured through a Western blotting assay. The expressions of Bax and Cleaved Caspase-3 were higher (Figures 7(a), 7(c), and 7(d)), and Bcl-2 expression was lower in the DM group than those in the Con group (Figures 7(b) and 7(d)). By contrast to the DM group, the GF group had further raised Bax and Cleaved Caspase-3 expressions, as well as further reduced Bcl-2 expression, implying that GF can induce apoptosis, conforming to the ultrastructural changes in TEM.

### 3.8. GF Increased Oxidative Stress in the Hippocampus of Diabetic Rats

Since GF restrained the Nrf2-HO-1/NQO-1 signaling pathway, the MDA level and SOD activity in the hippocampus were investigated more deeply. The results showed that the DM and GF groups had higher MDA level than the Con group, which was the highest in the GF group, signifying impairment by lipid peroxidation *in vivo* (Figure 7(e)). The SOD activity was attenuated in the DM and GF groups compared with that in the DM group, and it was the lowest in the GF group (Figure 7(f)), suggesting that GF impairs the antioxidant capacity and induces an unbalanced redox state in rats.

## 4. Discussion

There is growing evidence that people and animals with DM are more likely to suffer from cognitive decline than those without DM, but the exact mechanisms have not been fully identified. In addition, DM can arouse neurophysiological and structural changes in the brain, including amyloid deposition, hippocampal atrophy, and MRI signal abnormalities, which are collectively known as diabetic encephalopathy (DE) [29, 30], serving as an important factor for the increased risk of dementia. Compared to people with normal blood glucose, individuals with acute mild to moderate hypoglycemia may manifest reduced mental efficiency. Transient hyperglycemia has similar negative effects. The cognitive efficiency is decreased by about one-third under extreme blood glucose (either hypoglycemia or hyperglycemia) at a relatively mild level [31]. GF can lead to aberrant electrical signal activities in the left medial prefrontal cortex, which is correlated with the decrease in cognitive function score [32]. However, the specific mechanism by which GF causes these changes has not been clarified yet. The literature reports involving GF are mainly concentrated on clinical trials and *in vitro* cell experiments, and there are few studies on animal models. As for the modeling methods, 24 h satiety alternating with 24 h starvation, intermittent injection of glucose and insulin, and intermittent intake of a high-carbohydrate diet are primarily adopted. In the present study, an animal model of GF was successfully prepared through 24 h satiety alternating with 24 h starvation. It was uncovered that the blood glucose fluctuated wildly throughout the experiment. It is well known that diabetic patients and animals usually lose weight [33, 34]. It was found through this research that the weight gain in DM and GF rats flattened for 4-5 weeks after the increase of glucose, and the weight tended to decrease after week 5, which may be related to the glucose elevation and negative calorie balance *in vivo*. At the end of week 12, the body weight in the GF group was lower than that in the DM group, but the difference was not statistically significant. The reason is that when the rats in the GF group were fed again for 24 h, the food intake was increased significantly in the next 24 h due to starvation and hypoglycemia, which partially compensated for the weight loss caused by short-term caloric deficiency. Moreover, the body weight in the DM and GF groups rose overtly from week 5, which may be associated with the increased osmotic pressure due to elevated blood glucose, consistent with the conclusions from clinical practice.

As a gold standard of glucose control, HA1c is a pivotal player in the occurrence and development of diabetic complications. However, it merely represents the average glucose level instead of GF within 2-3 months. It has been revealed through the advancement of continuous glucose monitoring technology that as an indicator reflecting GF, time in range (TIR) is correlated with diabetic macroangiopathy, diabetic nephropathy, diabetic retinopathy, and cardiovascular disease. TIR ≥ 70% has been included in the Standards of Medical Care in Diabetes (2019) published by the American Diabetes Association (ADA) as a criterion for controlling T2DM, becoming a crucial supplement to HA1c [35]. In this study, the HA1c level in rats was significantly elevated in the DM and GF groups compared with that in the Con group, and it was lower in the GM group than in the DM group. On the contrary, the MWM test results corroborated that the rats in the GF group had poorer spatial learning and memory capacities than those in the DM group, suggesting that GF itself can lead to cognitive impairment independent of the impact of HA1c. It was reported that GF can contribute to cardiomyocyte apoptosis by triggering the endoplasmic reticulum stress pathway [19]. It was discovered that GF aggravates renal injury in diabetic rats by repressing the AKT signaling pathway [36]. However, few studies have been conducted on the relationship between GF and cognitive function changes. Totally, 121 elderly patients were enrolled for 48-hour continuous glucose monitoring, denoting that the mean amplitude of glycemic excursions (MAGE) has a correlation with the cognitive composite score, and such a correlation is independent of HA1c and fasting blood glucose [37]. According to our previous clinical studies, there is an association between TIR and Montreal Cognitive Assessment (MoCA) score; that is, patients with a low TIR level have a low MoCA score, implying that GF is an independent risk factor for cognitive impairment [38]. Nonetheless, the related mechanisms remain to be exploited.

The hippocampus is intimately related to learning and memory capacities [39–41], and increasingly, more evidence has demonstrated that the hippocampus is capable of causing phenomenological coherence of recollection by integrating different cortical representations with their space-time backgrounds *via* sparsely-connected neural coding. The processing of spatial scenes involves the parahippocampus. However, the right hippocampus is probably particularly implicated in the memory for positions in the environment, while the left hippocampus mostly participates in context-dependent episodic memory [42, 43]. GF can induce abnormalities in hippocampal sharp wave-ripples [44]. Studies have corroborated that the loss and necrosis of hippocampal neurons are the features of cognitive decline [45, 46], but there has been no report on whether GF exacerbates such situations and causes ultrastructural changes in hippocampal regions. Based on the results of HE staining combined with Nissl staining in this study, there were varying degrees of necrosis and loss of hippocampal neurons in the DM and GF groups, which were severer in the GF group. Furthermore, the observation of the ultrastructure by virtue of TEM manifested that GF could lead to neuron atrophy with a darker color, early apoptotic changes, and massive lipofuscin deposition. Lipofuscin, yellowish-brown microparticles accumulated in the cytoplasm, is the peroxidation product of unsaturated fats in organelle debris [47]. Free radicals can invade organisms to produce excessive MDA, further attacking the cells to stimulate lipofuscin deposition and causing cell injury. The aforementioned morphological changes coincided with cognitive impairment in the MWM test, suggesting that GF is more prone to accelerate hippocampal injury rather than constant hyperglycemia. Hence, the related mechanism was further investigated based on the morphological changes mentioned above.

Several studies have elucidated that wide variations in glucose levels may exacerbate hyperglycemia-induced oxidative stress [48–51]. Oxidative stress results from the imbalance of oxidizing substances and antioxidant defense substances *in vivo*, which is not only implicated in multiple pathophysiological processes but also highly correlated with various diseases such as neurodegeneration, DM, atherosclerosis, renal disease, osteoporosis, and tumors [52–55]. Nrf2 is a classical signaling pathway molecule capable of antagonizing oxidative stress. Nrf2 is dissociated from Keap1 under the stimulation of oxidative stress or inflammation, which enters the nuclei to increase the secretion of downstream HO-1 and NQO-1, thus enhancing the antioxidant capacity in the body. In living organisms, free radicals can trigger lipid peroxidation, with MDA as the final product, which can lead to crosslinking polymerization of proteins, nucleic acids, and other macromolecules and result in cytotoxicity. MDA content can reflect the degree of lipid peroxidation *in vivo* and indirectly manifest the severity of cell injury. As a vital antioxidant enzyme in living organisms, SOD is a natural enemy of oxygen free radicals, which can confront, block, and promptly repair the cell injury caused by oxygen free radicals. It was discovered through this study that hyperglycemia could inhibit the Nrf2 signaling pathway to decrease the downstream HO-1 and NQO-1, and GF was able to further repress the Nrf2 signaling pathway to exacerbate oxidative injury in the hippocampus. Besides, GF could significantly reduce SOD and raise MDA in rat hippocampus, thereby triggering the imbalance of oxidative stress.

*In vitro* experiments have verified that replacing the culture medium containing normal glucose with a high-glucose culture medium can facilitate the growth of neuronal cells and induce oxidative/inflammatory stress and microglial activation. In addition, the change from high glucose to normal glucose creates a metabolic stress state for microglia to induce cell apoptosis and autophagy, which can be corroborated by decreased Bcl-2, increased Caspase-3, and TUNEL staining [56]. Moreover, the GF-induced metabolic disorder in neuronal cells was simulated by exposing SH-SY5Y neuroblastoma cells to constant or fluctuating glucose for 24 and 48 h. It was uncovered that GF could attenuate the activity of mitochondrial dehydrogenase, suggesting that GF exerts a greater adverse effect on the energy regulation mechanism of neuronal cells than constant hyperglycemia or hypoglycemia [57]. Cell apoptosis, alternatively named as programmed cell death, is an active and noninflammatory death mode of organisms, during which phosphatidylserine eversion, mitochondrial membrane potential decline, Caspase activation, and DNA fragmentation occur commonly. Being an antiapoptotic protein, Bcl-2 consists of 4 homeodomains: BH1-BH4 [58, 59]. Acting as a proapoptotic gene, Bax can generate chain polymers under the irritation of apoptosis, then translocate from the cytoplasm to the mitochondrial membrane, increase the membrane permeability by interacting with pore proteins on the membrane, and trigger the release of cytochrome C from the mitochondria, thereby starting the Caspase-activated apoptotic signaling pathway [60, 61]. Initially cloned from human Jurkat T-lymphocytes, Caspase-3 is located downstream of the apoptotic cascade, which is called the executor of apoptosis. Caspase-3 is transformed into an active form (Cleaved Caspase-3) through clipping to perform its functions [62–64]. BDNF, a crucial member of the neurotrophic factor family with vital effects on the development of the nervous system, affects the growth, development, differentiation, and survival of neurons *via* multiple signaling pathways [65, 66]. The results of the present study elaborated that GF could further lower the Bcl-2/Bax ratio but increase the Cleaved Caspase-3 level in the hippocampus of diabetic rats, hinting that GF is capable of worsening the apoptosis of hippocampal neurons in diabetic rats, in agreement with the changes observed by TEM. Additionally, GF led to a further reduction of BDNF protein expression in the hippocampus of diabetic rats. These results explicitly illustrated that GF not only promoted oxidative stress injury and apoptosis in the hippocampal neurons of diabetic rats but also exacerbated cognitive impairment, which involved the suppression of the Nrf2 signaling pathway (Figure 7(g)). Therefore, other proteins in the Nrf2 signaling pathway will be detected continuously, Nrf2 inhibitors will be applied in future experiments, and other signaling pathways will be researched to probe into the exact mechanism in which GF aggravates cognitive impairment in diabetic rats.

This study has its obvious strengths. The small number of studies on the relationship between GF and cognitive dysfunction are mainly conducted in clinic, failing to explore the specific mechanisms; while in this study, an animal model of GF was established to observe the morphology and ultrastructure of hippocampal tissues, the indicators of apoptosis and oxidative stress were detected, and the proteins of cellular pathways involved were investigated. Thus, the findings of this study are of important significance for *in vivo* research on the influence of GF on the signaling pathways related to cognitive function and the relevant molecular mechanisms. The study, however, is also subject to several limitations: First, in addition to the Nrf2 pathway, other pathways are probably implicated in the mechanism by which GF affects the cognitive function, which may require further exploitation. Second, the Nrf2 signaling pathway was not validated, so it is planned to subsequently select rats with Nrf2 knockout for validation. Third, the GF group had exacerbated injury of hippocampal tissues compared with the group with constant hyperglycemia at the end of the experiment. However, the week ordinals of the experiment in which the cognitive impairment as well as the changes in histology and molecular biology occurs in the groups with GF and constant hyperglycemia, respectively, will be determined in future studies through tissues from rats sacrificed in different time periods.

## 5. Conclusion

GF can inhibit the Nrf2 signaling pathway to reduce the downstream HO-1 and NQO-1. Nevertheless, GF induces the increase of MDA plus the decrease of SOD and BDNF, thereby stimulating the loss, degeneration, and necrosis of hippocampal neurons in rats, aggravating oxidative stress and cell apoptosis, and facilitating DE progression. In clinical work, the fact that acute GF is more serious than chronic hyperglycemia should be concerned. Besides, endocrinologists should take measures to reduce GF while focusing on HbA1c, which may be conducive to reducing the occurrence of cognitive dysfunction.

## Figures and Tables

**Table 1:** Primers for qRT-PCR.

Genes	Forward primer (5′-3′)	Reverse primer (5′-3′)
Nrf2	ATTCCCAGCCACGTTGAGAG	TCCTGCCAAACTTGCTCCAT
HO-1	CTTCCCGAGCATCGACAACC	AATGTTGAGCAGGAAGGCGG
NQO-1	ATTGTATTGGCCCACGCAGA	GATTCGACCACCTCCCATCC
*β*-actin	CTCTGTGTGGATTGGTGGCT	CGCAGCTCAGTAACAGTCCG

## Data Availability

I can provide all raw data of this manuscript, if needed.
